# The Nuclear Receptor Field: A Historical Overview and Future Challenges

**DOI:** 10.11131/2018/101320

**Published:** 2018-07-26

**Authors:** Gisela I. Mazaira, Nadia R. Zgajnar, Cecilia M. Lotufo, Cristina Daneri-Becerra, Jeffrey C. Sivils, Olga B. Soto, Marc B. Cox, Mario D. Galigniana

**Affiliations:** 1 Departamento de Química Biológica, Facultad de Ciencias Exactas y Naturales, Universidad de Buenos Aires (1428), Argentina; 2 Instituto de Biología y Medicina Experimental- CONICET. Buenos Aires (1428), Argentina; 3 Department of Biological Sciences and Border Biomedical Research Center, University of Texas at El Paso, El Paso, TX 79968, USA

**Keywords:** Nuclear receptor, Steroid receptor, Chaperones, Heat-shock proteins, Hsp90, Transcriptional regulation

## Abstract

In this article we summarize the birth of the field of nuclear receptors, the discovery of untransformed and transformed isoforms of ligand-binding macromolecules, the discovery of the three-domain structure of the receptors, and the properties of the Hsp90-based heterocomplex responsible for the overall structure of the oligomeric receptor and many aspects of the biological effects. The discovery and properties of the subfamily of receptors called orphan receptors is also outlined. Novel molecular aspects of the mechanism of action of nuclear receptors and challenges to resolve in the near future are discussed.

## 1. Introduction

The largest group of transcription factors in eukaryotes is grouped in a superfamily of structurally related proteins referred to as the nuclear receptor superfamily. These receptors were first understood as ligand-regulated DNA-binding transcription factors, which regulate biological programs involved in a broad spectrum of physiological phenomena. With time, the so called ‘orphan’ receptors (receptors with no ligand discovered to date) were also characterized and included into this superfamily based on the structural domain properties of these transcription factors. It is currently accepted that in humans, the nuclear receptor superfamily comprises 48 members.

Nuclear receptors play several roles in the normal physiology of the cells, including metabolism, electrolyte balance, cell proliferation, immune response, enzyme activity, development, and reproduction, as well as in many pathological processes, such as cancer, neurologic and psychiatric syndromes, immunosuppression, diabetes, rheumatoid arthritis, asthma, hormone-resistance syndromes, cardiovascular diseases, metabolic syndrome, premature ageing, etc. [1–6]. Therefore, despite their already long history, these factors are still of great interest in modern biomedical research and drug discovery.

**Figure 1:**
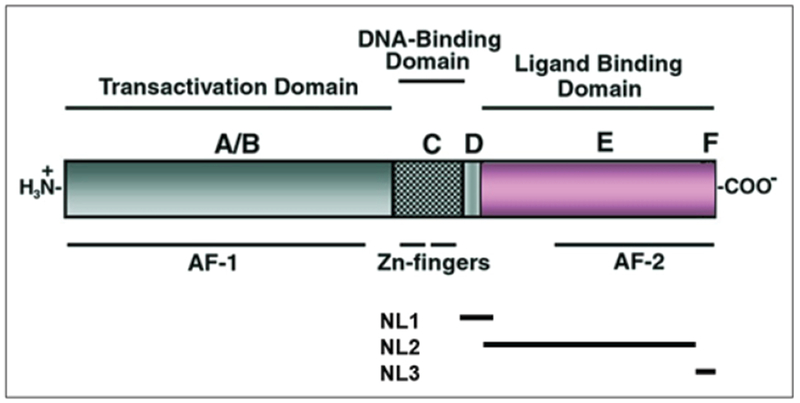
Domain structure of nuclear receptors (A-F). The DNA binding domain (DBD) or C region consists of two zinc-binding motifs (or Zn-fingers), that often includes a hinge domain or D region. A nuclear localization signal 1 (NL1) is usually located at the C-terminal end of this region. The ligand binding domain (LBD) or E/F region binds the cognate ligand. Ligands of the orphan receptor subfamily are not known, and it is thought that they could be endogenous compounds, possibly metabolic intermediates, or even environmental factors, which may explain some of their apparently constitutive transactivation activity and the difficulty encountered to identify their ligands. The AF1 and AF2 activation function 1 and 2 contact co-regulatory molecules, but AF-1 is typically a variable ligand-independent (first named transactivation domain. TD) while the AF-2 of the E/F region is ligand-dependent.

Even do the biological processes commanded by nuclear receptors is extremely vast, these proteins share remarkable structural similarity. Before the first genes encoding nuclear receptors were cloned, it was already known that they are modular proteins [7]. The greatest homology is preserved in the amino acid sequence of the DNA-binding domain (DBD) and the ligand-binding domain (LBD) [8, 9], in that order (Figure 1). As their names indicate, these domains are responsible for the association of the transcription factor with specific DNA sequences, the DBD, and the binding of small ligands, usually of lipophilic nature, the LBD. There is a third, non-conserved N-terminal domain named the transactivation domain (TD), which was first thought to be the most selective region of the protein due to interactions with other nuclear factors, consequently being responsible for the specificity of the biological effect (see [10–12] for comprehensive reviews). The TD, also named domain AF-1, shows variable length and sequence in the different family members and is recognized by coactivators and/or other transcription factors. Because of that variability, in the past it was also named the ‘immunoreactive domain’. The ability of the LBDs to activate transcription is controlled by the C-terminal helix 12, also termed AF-2, such that ligand-binding triggers a mechanism that transforms AF-2 into a transcriptionally competent domain. Nonetheless, today we know that those protein-protein contacts responsible for receptor specificity are not restricted to the TD only.

In the 1950s, the model for steroid hormone action told us that the steroid entered the cells by simple diffusion through the plasma membrane, after which a series of metabolic oxidations and reductions took place thus providing the ‘needed energy’ for growth stimulation and other specific actions. Subsequently, Elwood Jensen entirely overturned that elemental notion [13] when he proved that tritiated estrogens do not undergo chemical changes, but they rather bind to a protein within the cell. Then, this hormone-receptor complex must translocate to the cell nucleus and regulates the expression of specific genes. At that time, this idea was almost a heresy. “*That really got him into some hot water*,” recalled Gene DeSombre [14], Professor Emeritus in the Ben May Institute for Cancer Research, who worked with Jensen as a post-doctoral fellow and then as his colleague. “*Jensen struggled quite a lot*,“ echoed Shutsung Liao, another Ben May Institute colleague, who subsequently reported a similar mechanism for testosterone action. When Dr. Jensen first presented his data at the IV International Congress of Biochemistry in Vienna (1958), only five people attended to that session, three of whom were the other speakers. There were more than 1,000 attended registered to the meeting, but they attended to other simultaneous symposium on the metabolic processing of estrogen. Nonetheless, with this ‘trivial’ and totally unnoticed report, the concept of hormone receptor, as we understand it today, was born.

## 2. The Nuclear Receptor Superfamily

Before the genes encoding these receptors were cloned, the first member of the family to be identified biochemically was the estrogen receptor (ER) [15]. About two decades after, the cDNA for the human glucocorticoid receptor (GR) was the first to be elucidated [16]. followed by the ER [17] and the mineralocorticoid receptor (MR) [18]. Since then, nuclear receptors have become recognized as a superfamily of transcription factors, and steroid receptors were grouped as a subfamily within the former. Then, the nuclear receptor research field has undergone very rapid development and covers areas ranging from structural and functional analyses to the molecular mechanisms of transcription regulation. As soon as the founder members of the superfamily were characterized, a small group of non-steroidal receptors was also added to the family, i.e. the thyroid hormone receptors (TRs), the retinoic-acid receptors (RARs), and a relatively small legion of receptors whose endogenous ligands were unknown and were consequently grouped as the orphan receptor subfamily [8, 19].

Nuclear receptor genes are encoded and expressed even in the simplest organisms of the animal kingdom. It is accepted that more than 900 nuclear receptors genes have been identified in all animals examined [20], from the simplest to the most complex (NureXbase, http://nurexbase.prabi.fr/). The simplest metazoans belong to the phylum *Porifera*, for example sponges, which show primitive bodies with pores and channels allowing water to circulate through them. They have two genes encoding for nuclear receptors, whereas *Trichoplax adherens* (a flat organism of one millimeter in diameter, lacking any organs or internal structures), encodes for four receptors [20]. When the morphological and functional complexity of the organisms becomes more multifaceted, the number of receptors increases, reaching forty-nine members in mammals (the total number may vary if splicing variants are also counted). Nonetheless, it is pertinent to point out that nuclear receptors are absent in fungi, plants and also in the closest known relatives of metazoans, the free-living unicellular and colonial eukaryotes of the *Choanoflagellatea* class [21]. Hence, this evolutionary tree is telling us that these receptors should have arrived on the scene of evolution about 635 million years ago, i.e. when metazoans first appeared in the fossil records. Importantly, it seems that nuclear receptors play a cardinal role during the Cambrian explosion of life forms nearly 540 million years ago. Researchers have discussed for decades over what ignited that evolutionary burst. Some of them have postulated that a steep rise in oxygen sparked the change, whereas others say that it sprang from the development of some key, already still uncertain, evolutionary innovation. In this sense, it is interesting to pose the fact that the rising of the nuclear receptor family occurred during that time οCause or consequence? The precise reason has remained elusive and probably will be forever, mainly because so little is known about the physical and chemical environment of the planet at that time [22].

**Figure 2:**
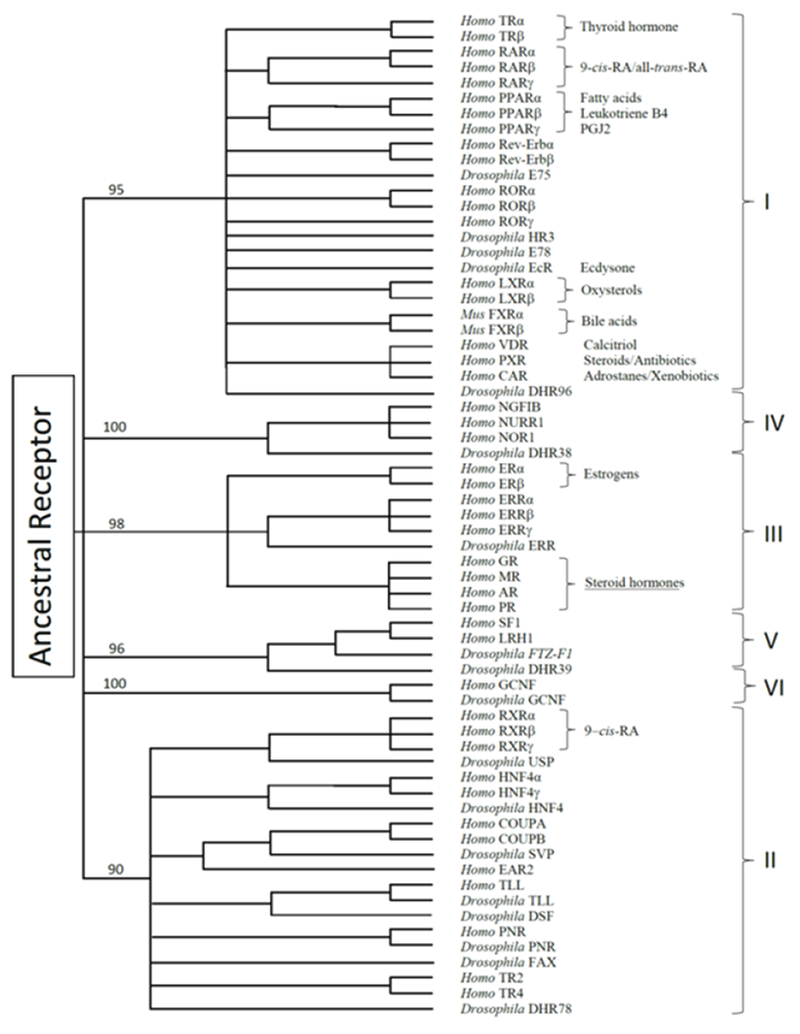
Evolutionary tree of the nuclear receptor family. The tree branches of the scheme show nodes well supported at bootstrap values >90%. Roman numbers group each subfamily of the superfamily. Note that the consensus phylogenetic position of the nuclear receptors is not correlated with the chemical nature of the cognate ligand.

Regarding the origin of the family, one possible hypothesis could be the notion that an ancestral nuclear receptor promoted ligands evolved independently in many lineages (Figure 2). An alternative theoretical possibility is that the ancestral receptor was indeed a protein designed for other biological purposes, but it gained specific functions upon ligand-binding in fortuitous event where preexisting small molecules, also designed for other purposes, began to activate these proteins in a typical gain-of-function event. It is difficult to affirm with absolute certainty what was first, the egg or the chicken. However, it is interesting to highlight that the same ligands used by these ‘novel’ receptors had appeared in nature well before than the proteins themselves. For example, the presence of progesterone in plants was first reported in the 60’s [23], and later detected in a wide range of plant species from 50 families [24]. Plant progesterone in turn, become a substrate precursor of corticosteroids, androstanes and estranes in this very same kingdom (see [25] for recent review). On the other hand, it is well known that there are mechanisms able to activate nuclear receptors in the absence of the cognate ligand [26–28], suggesting the possibility that ligands could have not been required during the evolution of this family.

Sequence alignment and phylogenetic tree construction resulted in a classification of the human nuclear receptor superfamily into six evolutionary groups of unequal size [29]: (I)- This large group contains the receptors TRs, RARs, VDR, all PPARs (peroxisome proliferator-activated receptors), and orphan receptors such as the Rev-Erb receptor, RORs (receptor tyrosine kinase-like orphan receptors), CAR (constitutive androstane receptor), PXR (pregnane X receptor), LXRs (liver X receptors), and others. (II)- This group includes RXRs (retinoid X receptor), COUP-TFII (COUP transcription factor II receptor), and HNF-4 (hepatocyte nuclear factor-4 receptor). (Ill)- This subfamily includes the steroid receptors and the ERRs (estrogen-related receptors). (IV)- This group contains the nerve growth factor induced clone B group of orphan receptors (NGFI-B, NURR1, and NOR1). (V)- This small group that includes the steroidogenic factor 1 (NR5A1) and receptors related to the Drosophila FTZ-F1. (VI)- This subfamily contains only the germ cell nuclear factor-1 (GCNF1) receptor, which does not fit into any other subfamily.

### 2.1. Steroid receptor subfamily

As it was commented before, the early beginning of the nuclear receptor superfamily is traced back to the late 50’s when tritiated-estrogens were used by the first time in studies on the biochemistry of steroid receptors [13, 30]. By using this ‘novel tool’, it was demonstrated the selective accumulation and retention of [^3^H]-labeled steroid in the reproductive organs of immature female animals when physiological amounts of hormone were injected. Therefore, it was postulated that the retention of [^3^H]-estradiol in the uterus and vagina reflected binding to putative receptors located in the cells [15], this being the first arguable evidence for binding of a hormone to a receptor. With time, several members of the steroid receptor family were cloned during the ‘80s, such as the glucocorticoid receptor [26], the estrogen receptor [27], the thyroid hormone receptor [28], and the mineralocorticoid receptor [29].

Studies on maximum likelihood sequences of ancestral receptors and branch lengths were reconstructed on the most parsimonious phylogeny [31]. The hypothesis by Joseph Thornton was based on the fact that an ancestral protein is likely to have been most similar in sequence and therefore in function to the descendant gene that diverged more slowly after the duplication event. The observed sequence divergence rate suggested that the ancestral steroid receptor was a functional estrogen receptor. Therefore, it is currently thought that ER was the first receptor, followed by the PR. Similarly, the youngest members of this subfamily are GR and MR, in that order [31].

Also, the appearance of puffs on the polytene chromosomes of insect salivary glands incubated with 20-hydroxyecdysone provided the first evidence that steroids may act directly at the gene transcriptional level to bring about subsequent cellular changes [32, 33], In line with these findings, early studies also showed that estrogen can selectively activate the genes encoding egg-white and yolk proteins. The first attempts to characterize those [^3^H]estradiol-bound proteins in native tissues were performed during the middle ‘60s when Toft and Gorski [34] prepared cytosolic fractions from uteri of rats injected with [3H]estradiol and resolved its components through a gradient of 5–20% sucrose. The estradiol radioactivity sedimented in a symmetrical peak at 9.5S, which disappeared after protease treatment. Shortly thereafter, it was shown [35] that the direct addition of [^3^H] steroid to cytosol extracts obtained from uteri of untreated rats also yielded identical 9.5S complexes. This was the first direct demonstration of steroid-binding to a receptor in a cell-free system, and set the basis to establish the so called “untransformed”, 9.5*S* isoform of the receptor versus the “transformed” isoform evidenced at 4-5S at higher temperatures than 2-3°C or by increasing the ionic strength of the buffer. The term “transformation” began to be employed to identify the conversion of the 9.5S, non-DNA-binding isoform of the receptor, to the 4S, DNA-binding form, which is demonstrated after the dissociation of the associated chaperone heterocomplex. On the other hand, the term “activation” was used to specifically refer to the conversion of receptors from a form that does not bind steroid to a steroid-binding form.

In spite of the overwhelming evidences related to the existence and roles of steroid receptors, as late as in 1968 the use of the term “receptor” was still questioned by some researchers, as it can be read in articles published in prestigious journals, where it was even argued that the steroid-binding macromolecules “*may be without physiologic significance*” [32]. Curiously, the concept of steroid receptor evolved in parallel with the discovery of close-associated proteins that are essential for receptor folding and function—the molecular chaperones. Notably, the biological relevance of these proteins was also questioned by that time.

### 2.2. Molecular chaperones

From the etymologic perspective, the term *chaperone* refers to a person (usually a matron) who used to accompany young unmarried women in public to supervise them at a social gathering. Therefore, a chaperone person was a ‘social protector’ who safeguarded the proper conduct of teen-agers. Analogously, proteins that assist others in their proper folding are also referred to as ‘chaperones’, i.e. Hsp90, Hsp70, CyPA, TRiC/CCT, Grp94, GroEL/GroES, etc. The term ‘molecular chaperone’ was first used to describe the ability of nucleoplasmin to prevent the aggregation of histones with DNA during the assembly of nucleosomes, and then, it was extended to other proteins that mediate the post-translational assembly of protein complexes (see an appealing background in [36]). Moreover, those molecular chaperones induced by heat-stress are also named heat-shock proteins (HSPs). Nonetheless, temperature is not the only stimulus able to trigger such a biological response, but also UV radiation, chemicals, toxic compounds, metals, inappropriate pH or osmotic pressure, nutrient starvation, oxidants, fever, cancer, infections, neurodegenerative diseases, etc. [37, 38]. Also, the heat-shock response is useful even in the absence of stress during the normal growth cycle of the cells even without the existence of stressors in the medium. Importantly, while all HSPs are molecular chaperones, not all chaperones are always induced by heat-shock or other types of stress. Therefore, the level of expression for the latter group is quite stable. On the other hand, the term ‘*co-chaperone*‘ refers to proteins that are associated to chaperones assisting them in their properties to modulate client-proteins properties (for example, TPR-domain immunophilins, p23, Ahal, CDC37/p50, Hop/p60, SGT, etc.). This does not imply that a co-chaperone cannot show chaperone properties, which depends on the client protein and the abundance of other factors.

The biological importance of HSPs can be traced back to the same period the concept of receptor was born, the early 1960s. The Italian scientist Ferruccio Ritossa was studying the synthesis of nucleic acid in puffs of *Drosophila* salivary glands. A colleague accidentally changed the temperature of the cell incubator and something unexpected was noticed—an incredible transcriptional activity of new chromosomal puffs. New RNAs were detected as soon as to 2-3 nun after increasing the temperature. The importance of this fortuitous observation was immediately grasped—cells react in response to elevated temperature through the synthesis of unknown factors [39]. Today, we know that these factors are the HSPs, and this finding remains to date as the clearest demonstration of environmentally induced changes in gene expression. As it often happens, this unanticipated concept was very difficult to accept at the time of discovery. Ritossa’s fortuitous but clever observation was systematically rejected from top journals with the argument that, again, “*the finding is interesting, but it lacks biological relevance*” [40]. Today anyone could even think about the possibility to question the biological relevance of chaperones in protein folding, client protein stability and biological function. Moreover, the further evolution of knowledge of both the steroid receptor field and the chaperone field became close-related one another. In the particular case of the chaperone heterocomplex associated to steroid receptors, it is not only essential to favor steroid binding and to prevent receptor transcriptional activity, but also for the stabilization of the receptor conformation preventing its degradation by the proteasome, as well as it is also a critical component of the molecular mechanism of transport and subnuclear redistribution of these receptors [41–43], as well as other factors [44–47].

## 3. Receptor Transformation

Initially, laboratories focused their studies on the capability of transformed cytosolic receptors to bind to nuclei. Several assays for receptor transformation were available, such as the finding that treatment of nuclei with DNAse released transformed receptors, and it was shown that transformed receptors are able to bind polyanions in general. Researchers took advantage of these observations and assays of receptor transformation were developed based on binding to phosphocellulose, ATP-Sepharose, and carboxymethyl-Sephadex.

The Gustafsson laboratory [48] was the first to combine steroid- and temperature-dependent transformation of the GR to the DNA-binding state as a first step to enrich preparations to purify receptor. This allowed to define by the first time by limited proteolysis the ligand binding domain (LBD) and the DNA-binding domain (DBD) [49], well determined years after when the receptor was cloned. The delimitation of these domains was useful in later studies localizing the Hsp90-binding region on molybdate-stabilized untransformed receptor [50]. By that time, it was well known that Hsp90 was a highly conserved and wide distributed chaperone in all organisms, located mostly in the cytoplasm, and phosphorylated mostly on serine residues. A 90-kDa protein with the same characteristics had been reported bound to the untransformed receptor [51]. Three independent studies reported by 1985 proved that that 90-kDa protein was the chaperone Hsp90 [52–54]. By co-immunoadsorption assays, it was demonstrated that Hsp90 dissociated from the GR when cytosolic receptors were transformed, a phenomenon that was prevented by molybdate [55]. Subsequent to these first studies where PR and GR oligomeric complexes were characterized, Hsp90 was demonstrated to be present in all other untransformed members of the steroid receptor subfamily, ER, AR, MR, and VDR (vitamin D receptor), as well as in other members of the nuclear receptor superfamily such as Rev-Erb, AhR, PPAR*γ*, CAR, etc., but not in TR, RAR, RXR, and some members of the orphan receptor subfamily.

Hsp90 is a highly conserved HSP expressed ubiquitously in all organisms. It is the most abundant constitutive HSP accounting for ~1-3% of the total cytosolic proteins of the cell. Two genes encode Hsp90 in mammalian cells, where the Hsp90α form shows 86% identity with respect to Hsp90*β* [56]. Moreover, there is extensive homology with lower species; thus, the human Hsp90*β* shows 78% identity with the *Drosophila* 83-kDa HSP, and 61% identity with yeast Hsp90 [57]. In *Escherichia coli*, the homologous HSP is a 63-kDa protein 42% identical to human Hsp90 [58]. In the mouse, there is a clear difference in the electrophoretic migration of both forms of the protein on denaturing gels, and the α and *β* forms are often called Hsp86 and Hsp84 respectively, with Hsp84 being more abundant than Hsp86. By coimmunoadsorption, it was demonstrated that Hsp86•Hsp84 heterodimers exist as native complexes [59]. In addition to being expressed at a high level in normal cells, Hsp90 is heavily phosphorylated even in the absence of stress and it migrates as several species on two-dimensional gels [60].

As it is expected for any chaperone, Hsp90 facilitates the proper folding of proteins, but it also provides biological activity to client proteins that still preserve an intact tertiary structure acting like a biological switch, becoming essential for various cellular processes, such heterocomplexes assembly, protein degradation, signal transduction cascades, and morphological evolution [61–63]. Hsp90 is commonly located in the cytoplasm, but a small fraction of Hsp90 is also present in the nucleus, in particular when cells are exposed to stress. Stability of various oncogenic factors is almost entirely dependent on Hsp90 binding, such that cancer cells require this chaperone to survive in the demanding milieu generated by oncogenic transformation. Consequently, Hsp90 has become an attractive antineoplastic drug target, and several Hsp90 inhibitors are being currently tested in various stages of clinical trials [64–66].

During the early ‘90s, it was shown that Hsp90-based heterocomplexes could be assembled in vitro with client factors by incubating immunopurihed receptor or protein kinase with rabbit reticulocyte lysate and a source of ATP [67, 68]. Most of the advances in this regard were achieved by similar studies performed in parallel with the GR and PR. These reconstitutions could also be achieved by using purified proteins [67, 69, 70], such that the 9S, untransformed complex, could be rebuilt. These types of studies permitted the elucidation of the sequential assembly cycle of the Hsp90 heterocomplex with the GR, one of the best characterized complexes. Thus, it was demonstrated that receptor complexes are assembled in a specific order and dynamic manner, the first step being the formation of an (Hsp90)_2_•Hop•Hsp70•Hsp40 oligomer, which is stabilized by the presence of the Hsp90-binding co-chaperone p23. Today we know that the stabilizing action of p23 mimicked that action first assigned to the addition of molybdate to buffer preparations, which in turn restricts the nuclear accumulation of GR.

The TPR-domain co-chaperone Hop is finally released from Hsp90, and its site is occupied by a TPR-domain co-chaperone, usually FKBP51, FKBP52, PP5, or Cyp40, which dynamically exchange on Hsp90 dimers bound to untransformed, 9S receptors [71–73]. Studies of saturation binding of Hop to Hsp90 dimer [74] and cross-linking of Hsp90•FKBP52 complexes [75] are consistent with one TPR acceptor site per Hsp90 dimer, so the relative expression of a given TPR protein in the cell may be proportional to the extent of such protein present in the untransformed complexes [44,45,64,76]. Even so, the role of the steroid hormone is also quite relevant. Recent evidence showed that aldosterone-binding to MR favors the exchange of FKBP51 for FKBP52, whereas the synthetic agonist 11,19-oxidoprogesterone favors the recruitment of PP5 [71]. From the perspective of receptor trafficking, both FKBP52 and PP5 are equally effective for MR retrotransport because they associate dynein in similar extent [77]. The qualitative composition of the untransformed form of the receptor may vary according to the nature of the receptor. Thus, some immunophilins such as CyP40 shows selective preference for the ER rather than the GR or MR, whereas FKBP52 shows preference for GR and PR, and not for AhR, which in turn recruits exclusively XAP2 and not the other TPR-domain immunophilins.

## 4. The Subfamily of Orphan Nuclear Receptors

During the decade 1988 to 1998, there was an increased research focus on the nuclear receptor superfamily. By using known and naturally occurring ligands, receptors such as ER, AR, GR, TR and VDR became what are now nuclear receptors, whose function and ligands were well identified [78–80]. Insight into structural similarities and conserved domains amongst steroid hormone receptors paved the way for the discovery and addition of new members into the nuclear receptor superfamily [80]. In contrast to the historically named “classic receptors”, the newly introduced family members lacked an established partnership to endogenous ligands, and thus were designated as “orphans”.

With the advent of molecular techniques and the generation of cDNA libraries, members of the superfamily were discovered to share a conserved domain arrangement consisting of the modulator region (A/B) with an activation function 1 (AF1) domain, a DBD, a hinge region, a LBD, and, within this domain, an activation function 2 (AF2) region [78, 79, 81, 82]. The information derived from the sequences and conserved regions of known nuclear receptors enabled the development of molecular probes that could be used to screen cDNA libraries from various tissues, and led to the identification of additional orphan receptors. This time period came to be known as “the genomic era” [83].

In 1988, using the sequence of the DNA-binding domain from the ER, the first two orphan nuclear receptors were discovered, which included Estrogen Related Receptor α (ERRα) and Estrogen Related Receptor β (ERRβ) [84]. Likewise, following a screen using a mouse liver cDNA library, and with the belief that chemicals known as peroxisome proliferators (PP) may act via nuclear receptors similar to that of the steroid hormones. Peroxisome Proliferator-activated receptors (PPARs) were added to the family in the early 1990’s [85, 86].

With the continuous discovery of orphan receptor superfamily members, research goals steered to the search for potential receptor ligands, which was achieved with the use of “reverse endocrinology”. Reverse endocrinology uses the receptor itself to screen for ligands, as opposed to using known ligands to find receptors [87]. This methodology not only allowed for the screening of potential endogenous ligands, but also any additional compounds, natural or synthetic, acting as ligand partners for the orphan receptor in question. When an endogenous ligand for an orphan receptor is identified, the receptor is no longer an orphan, and it is placed into the “adopted” orphan receptor family.

The first endogenous ligand established for one of the orphans was 9-*cis*-retinoic acid, moving the retinoid X receptor (RXR) from the orphan family to the adopted family. By the end of 1998, ligands had been discovered for 13 orphans, including but not limited to PPAR, LXR, and PXR [88–91]. Moreover, once a ligand has been identified for a given orphan receptor, that information can be used to assist in determining an orphan receptor’s physiological function(s), and discerning new physiological pathways in which a specific receptor plays a role. In light of the fact that all NRs play significant roles in the regulation of human physiology, establishing the ligands that activate orphan receptors can open the door to the development of novel therapeutic strategies for diseases [78, 79]. Even though identification of endogenous ligands for orphan receptors is crucial, adoption of orphan receptors is not completely achieved until said ligands have been validated under physiological conditions. To date, there is a lack of consensus for the possible ligands of most members of the orphan NR superfamily. Adopted or true orphan NRs, such as PPAR, LXR, PXR, and Nurr1, have shown potential as therapeutic targets in the treatment of diabetes, obesity, and neurodegenerative diseases.

Of the previously mentioned orphan nuclear receptors PPAR, LXR, FXR, and PXR have been adopted, yet controversy still remains due to the relatively low affinity binding of their identified interacting ligands (micromolar range), as well as the promiscuous interactions of these receptors. However, the low affinity binding was also the first sign that not all nuclear receptors could need high binding-affinities for their ligands. Today we know that even for the case of formerly named classic receptors (ER, RAR, VDR, AR, etc.), our first understanding of their interaction with cognate ligands could have been an oversimplification since some receptors are activated only after physiological levels of their ligands have been exceeded. Moreover, local concentrations in some tissues (particularly, in the nervous system) hormone levels could largely exceed the values of an “acceptable” *Ka* value. Moreover, ligands first thought to be side metabolites of the normal metabolism of agonists such as allopregnanolone (or tetrahydroprogesterone) became biologically active in several ways; thus, in addition to be a known GABA_*A*_ receptor agonist, allopregnanolone has recently been demonstrated to activate PXR-dependent pathways in the *μ*M range [92]. Thus, certain bile acids (e.g., lithocholic acid) have been shown to directly activate PXR at concentrations between 10 *μ*M to 100 *μ*M [93], Moreover, even though three bile acid precursors (7α-hydroxy-4-cholesten-3-one, 5*β*-cholestan-3α, 7α,12α-triol, and 4-cholesten-3-one) activate mouse PXR in the low *μ*M range, the same ligands are less potent activators of its human ortholog [94]. This type of differences for ligand specificity also extends to other xenobiotic ligands [95].

Other nuclear receptors, which were once orphans and are now adopted, are the xenobiotic sensors CAR and PXR. These receptors participate in the response against xenobiotics, and are known to facilitate the excretion of toxic metabolites from both endogenous and exogenous sources [98]. CAR and PXR were initially identified as xenosensors that regulate the expression of phase I and II xenobiotic metabolizing enzymes, and are known to be activated by common ligands such as ethinyl estradiol, diethylhexyl phthalate, and clotrimazole [86]. Remarkably, CAR and PXR have structurally distinct properties from those of other members of the family. PXR has the ability to bind ligands with varying sizes due to its flexible ligand binding domain; while CAR is constitutively active partly due to its rigid ligand domain, which might reduce the ability of some ligands to activate the receptor [99]. Taken together, PXR and CAR demonstrate the ability to influence drug metabolism, and the potential to shape current treatments owing to recently discovered knowledge on environmental and dietary chemicals that alter their activity.

**Figure 3:**
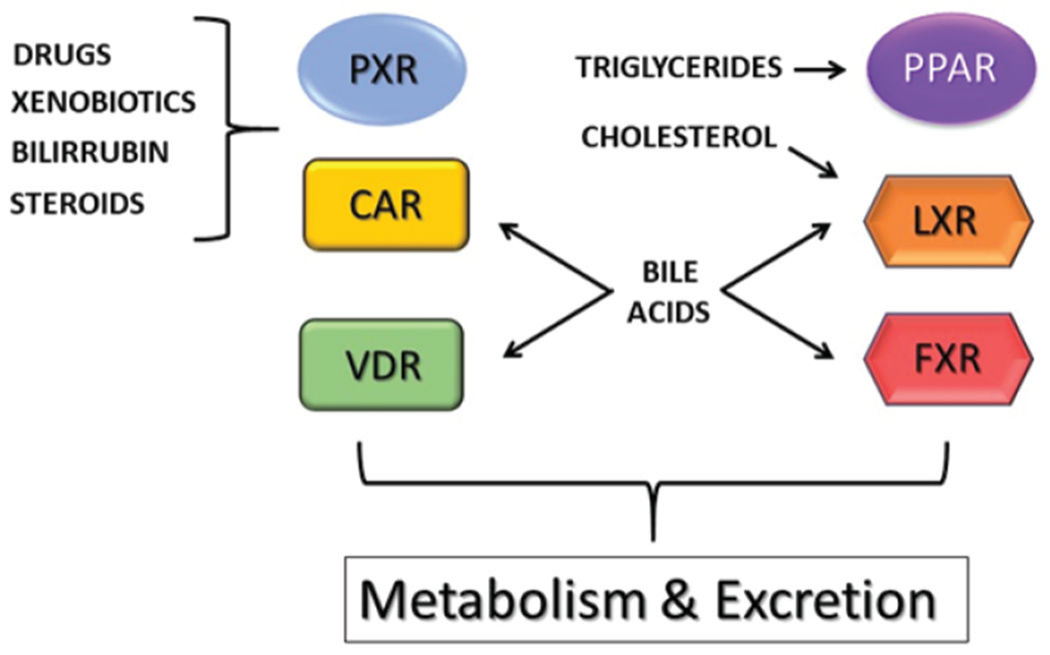
Endogenous and xenobiotic lipophilic ligands. Various xenobiotics and endogenous lipids are able to activate several nuclear receptors, which in turn control have the physiological role of controlling the intrahepatic and extrahepatic levels of these compounds. Thus, these receptors regulate the metabolism and excretion of these compounds. Note that a relatively high redundancy exists for several substance classes to bind to multiple receptors.

Originally, ligands of the PXR and CAR receptors were exogenous drugs and xenobiotics, 1α,25-dihydroxyvitamin D3 for VDR, oxysterols for LXR, and bile acids for FXR (see Figure 2). However, later findings showed that a number of endogenous compounds are also able to influence PXR and CAR activity and that these xenosensors share an overlapping ligand pattern with other members of the subfamily (see Figure 3). The overlap of endogenous lipids to activate CAR, PXR, FXR, and LXR indicates a functional connection between these receptors in liver physiology [96].

On the other hand, PPAR was initially named after a lipid-lowering drug that causes peroxisome proliferation was able to activate the receptor. The three known PPAR subtypes are PPARα, PPAR*β*, and PPARδ, which help support fatty acid oxidation, and PPAR*γ*, which aids in maintaining glucose homeostasis by increasing insulin sensitivity [97]. These receptors have been regarded as promising therapeutic targets in the treatment for obesity and diabetes. Similarly, LXRs have been shown to play a significant role in glucose homeostasis and metabolism by upregulating glucokinase and promoting glycogen synthesis [97]. Furthermore, LXRs respond to elevated levels of sterols to minimize buildup of cholesterol in the liver [80]. LXR and PPAR demonstrate the importance in human disease progression of establishing endogenous ligands to orphan receptors. Ultimately, the use of specific ligands for LXR and PPAR also offer an interesting and novel approach to treat atherosclerosis and type II diabetes, respectively [83, 91].

Much like CAR, Nurr1, from the NR4A subfamily of nuclear receptors, is structurally different from the other members of the superfamily. Although the ligand binding domain of Nurr1 is noticeably similar to that in any other nuclear receptor, it does not contain a cavity for ligand binding [100]. Therefore, Nurr1 cannot rely on a cognate ligand for its regulation, but rather by alternative mechanisms, leaving Nurr1 as an orphan NR. Nurr1 is expressed primarily in the central nervous system and is known to be essential for the development, survival, and migration of dopaminergic neurons [83, 101]. Because Parkinson’s disease (PD) results from the loss of dopaminergic neurons, Nurr1 is suggested to play a role in pathogenesis of PD [83]. Not surprisingly, Nurr1 mutations have been linked to PD and dysregulated Nurr1 expression has been observed in PD midbrains [102]. Despite a lack of well-defined regulatory partners and the challenge this poses for disease treatment, Nurr1 demonstrates great potential as a therapeutic target for PD treatment options.

Identifying biological functions and physiological ligands that activate orphan receptors has broadened our knowledge about a variety of diseases implicated in activation of these receptors. Further dissecting signaling networks and the presence or absence of physiological ligands is of utmost importance to promote our understanding of disease progression involving orphan receptors.

Based upon phylogenetic analysis, the unified nomenclature system divides the nuclear receptor superfamily into six structural and distinct groups [86, 103]. The chronological discovery of orphan receptors in order of the discovery date of the first orphan in each family, the discoverer and adopted ligand determinations are summarized in Table 1.

## 5. Future Challenges

Given the wide variety of processes controlled by nuclear receptors, their dysregulation usually contribute to the development and/or progression of numerous diseases, including cancer, immunosuppression, diabetes, infertility, pharmacologic intolerance, etc. Because most of these receptors bind small molecules able to regulate their biological activity, these receptors also represent promising therapeutic targets for which selective agonists and antagonists can be engineered [104]. In view of the fact that nuclear receptors regulate many genes in several tissues, novel synthetic ligands usually show beneficial therapeutic effects and unwanted side effects that limit clinical use. Major goals in the nuclear receptor field therefore include attaining a better understanding of the mechanisms underlying their actions in specific cell types and the ways in which these receptors selectively modulate their activities.

Selective nuclear receptor modulators also show partial agonist-antagonist activity. Thus, ligands with such particular features have been developed for a number of NRs, such as ER, AR or PPARs [105]. These properties are associated with differential recruitment of coactivators and corepressors and the tissue-selective expression profiles of these coregulators. A similar proposal has been made for the regulatory Hsp90-binding immunophilins that regulate steroid receptor and NF-kB function [45], Interestingly, there are cases where subtype selectivity and response element selectivity overlap in quite complex manners. A good example is the case of the ER ligand raloxifene, which acts as an antagonist of estradiol at a simple estrogen response element for both subtypes of the estrogen receptors, ERα and ER*β*. However, at an AP1 response element the same ER ligand behaves like an agonist with the ER*β* subtype, estradiol being an antagonist [106]. Binding studies evidence no difference in the affinity constant of raloxifene to either receptor subtype, leading to the conclusion that the differences must involve something subtler at the transactivating surfaces of the two ER subtypes at an AP-1 element.

Drugs that target nuclear receptors are to date among the most widely used and commercially successful. For example, bexarotene and alitretinoin (RXRs), fibrates (PPARα), and thiazolidinediones (PPARγ) are drugs approved for treating cancer, hyperlipidemia, and type 2 diabetes, respectively [107]. Looking out on the event horizon of small ligand discovery for nuclear receptors, it is noteworthy that LXR and FXR agonists are in development for treating non-alcoholic steatohepatitis and preventing atherosclerosis [108]. Perhaps just as importantly, PXR is now used routinely in the pharmaceutical industry to screen all new drug candidates for potentially dangerous drug-drug interactions. In line with this, the RXRs and similar receptor partners will become the next generation of relevant therapeutic targets.

From an evolutionary perspective, remarkable advances have been made in the field of nuclear receptors and their cognate ligands sheading light in our understanding of the evolutionary origins of life. This allowed a new view of nuclear receptors and their ligands to emerge. Clearly, the endocrine system is an issue of evolution that has prompted today’s biochemists to revise the old hypothesis that the hormone and its receptor could have been preexisting structures, the interaction of their corner stones being necessarily the result of evolution itself. Indeed, the information for hormonal regulation is written not only in the hormone structure, but also in the receptor, so that both components function as a unit. In higher organisms, the nuclear receptor superfamily bears a close resemblance to its primordial predecessor. On the other hand, signaling molecules seem to have acquired their present role in a long evolutionary process, which may well sharp the separation between close nuclear receptors partners such as GR and MR. This view of the modern day endocrine nuclear receptors evolving from more ancient receptors that originally sensed their environment or ligands that were first used for other purposes is not only consistent with the early events in the history of life in the Earth, but also with the prevalence of environmental signals that may act as endocrine disrupters via nuclear receptors. Similarly, these ancient properties could also be used to take advantage for the design of novel ligands that resemble those lost or attenuated biological activities.

## Figures and Tables

**Table 1:** The Orphan Receptor Subfamily.

Unified Nomenclature	Common Abbreviations	Date Identified	Discovered By	Ligand
NR3B	Nreb1/ERRα	1988	[84]	

	Nr3b2/Errβ	1988	[84]	

	Nr3b3/Errγ	1998	[109]	

NR4A	Nr4a1/Nur77 (*Ngfi-b*, *Tr3*, *N10*, *Nak-1*, *St-59*),	1988	[110, 111]	

	Nr4a2/Nurr1 (*Rnr-1*, *Not*, *Tinur*)	1992	[112]	

	Nr4a3/Nor1 (*Tec*, *Minor*, *Chn*)	1995	[113]	

NR2F	Nr2f2/Coup-tfII	1988	[114]	

	Nr2f6/Ear2	1988	[114]	

	Nr2f1/Coup-tfI	1989	[115]	

NR2C	Nr2c1/Tr2	1988	[116]	

	Nr2c2/Tr4	1994	[117]	

NR1D	Nr1d1/Rev-erbα	1989	[118]	Heme [119–122]

	Nr1d2/Rev-erbβ	1994	[123]	

NR2B	Nr2b1/Rxrα	1990	[90]	9-CIS retinoic acid as well as interacting with other NR [89, 124–127]

	Nr2b2/Rxrβ	1991	[128]	

	Nr2b3/Rxrγ	1991	[129]	

NR2E	Nr2e1/T1x	1990	[130]	

	Nr2e3/Pnr	1999	[131, 132]	

NR1C	Nr1c1/Pparα	1990	[85]	Fatty acids [133, 134]

	Nr1c2/Pparβ or δ	1993	[135]	

	Nr1c3/Pparγ	1993	[135]	

NR5A	Nr5a/Sf1	1992	[136]	Phospholipids [137]

	Nr5a2/Lrh1	1992	[138]	

NR1F	Nr1f1/Rorα	1993	[139]	Sterols ?

	Nr1f2/Rorβ	1993	[139]	

	Nr1f3/Rorγ	1996	[140].	

NR2A	Nr2a1/Hnfα	1994	[141]	Fatty acids ? [142]

	Nr2a2/Hnfγ	1996	[143]	

NR1I	Nr1i3/Car	1994	[144]	Xenobiotics and endobiotic [79]

	Nr1i2/Pxr	1998	[145–148]	

NR6A	Nr6a1/Gcnf	1994	[149]	

NR0B	Nr0b1/Dax-1	1994	[150]	

	Nr0b2/Shp	1996	[151]	

NR1H	Nr1h2/Lxrβ	1995	[152]	Oxysterols and bile salts [78, 79]

	Nr1h4/Fxrα	1995	[153]	

	Nr1h3/Lxrα	1995	[152]	
